# In Vitro Assessment of Ozone-Treated Deoxynivalenol by Measuring Cytotoxicity and Wheat Quality

**DOI:** 10.3390/toxins16020064

**Published:** 2024-01-25

**Authors:** Chao Sun, Chuncai Mao, Zhie Zhou, Jianhui Xiao, Wenwen Zhou, Juan Du, Jun Li

**Affiliations:** 1 College of Food Science and Engineering, Jiangxi Agricultural University, Zhimin Avenue, No. 1011, Nanchang 330045, China; jackssunc@jxau.edu.cn (C.S.); wenwenzhou@jxau.edu.cn (W.Z.);; 2 Jiangxi Enterprise Technology Center, Huangshanghuang Group Food Co., Ltd., Xiaolanzhong Avenue, No. 66, Nanchang 330052, China

**Keywords:** deoxynivalenol, assessment, in vitro toxicity, cytotoxicity tests, wheat quality

## Abstract

Deoxynivalenol (DON), a trichothecene mycotoxin, could lead to cytotoxicity in both animal bodies and plant seed cells. Ozone degradation technology has been applied to DON control. However, the safety and quality of the contaminated grain after DON degradation are largely obscured. In this work, we evaluated the cytotoxicity of ozone-treated DON through seed germination experiments and cytotoxicity tests. Cell experiments showed that the inhibition rate of HepG2 viability gradually increased within the concentrations of 1–10 mg/L of DON, alongside which an IC50 (half maximal inhibitory concentration) of 9.1 mg/L was determined. In contrast, degrading DON had no significant inhibitory effect on cell growth. Moreover, a 1–10 mg/L concentration of DON increased production of a large amount of reactive oxygen radicals in HepG2, with obvious fluorescence color development. However, fluorescence intensity decreased after DON degradation. Further, DON at a concentration of >1 mg/L significantly inhibited the germination of mung bean seeds, whereas no significant inhibition of their germination or growth were observed if DON degraded. Changes in total protein content, fatty acid value, and starch content were insignificant in wheat samples suffering ozone degradation, compared to the untreated group. Lastly, the ozone-treated wheat samples exhibited higher tenacity and whiteness. Together, our study indicated that the toxicity of DON-contaminated wheat was significantly reduced after ozone degradation.

## 1. Introduction

Deoxynivalenol (DON) is a fungal and widely contaminating toxin mainly produced by *Fusarium graminearum*, notorious as the most common cereal toxins found in wheat, corn, and barley [1,2]. The level of DON needed to exceed maximum limits in feed is higher than in food [3]. DON inhibits cell growth and protein synthesis and causes DNA and RNA damage [4]. DON could contribute to remarkable cytotoxicity on HepG2 at an IC_50_ of 4.73 mg/L (in human liver cancer cells), within a broad concentration and time range [5]. DON could affect the development of HT29 intestinal cancer cells, with changes in protein expression and cell signaling pathway transduction [6]. Mycotoxin DON could cause cell death in wheat (*Triticum aestivum*). On the contrary, it reduces death of *Arabidopsis thaliana* if encountering heat shock [7].

Ozone is an unstable strong oxidizing agent that can quickly decompose and destroy microorganisms and organic matter in water [8,9]. It has been applied in food storage, sterilization, and degradation of mycotoxin [10]. Our previous studies have found that toxin contents (15 mg/L of DON) were significantly reduced after ozone (ozone at 80 mg/L) degradation [11]. However, a few studies have been conducted on quality changes during ozone degradation of wheat contaminated by DON. The main nutrients in wheat are starch (70%), protein (11%), and fat (1.6%) [12]. In particular, the determination of the effects of ozone-degrading DON on these three nutrients in wheat is urgently required. No significant detrimental changes in the starch pasting-properties of DON-contaminated wheat are caused by ozone, and there was a slight rise in the dough development time and stability time, suggesting that the quality of flour may improve after ozone treatment [13]. Moreover, plant seeds such as mung bean, soybean, and peanut are very sensitive to DON toxin, and their germination is inhibited at low concentrations [14,15]. In order to further analyze the role of ozone degradation of DON in monitoring cytotoxicity and grain quality, in vitro assessments related to cell experiments [16] and plant seed germination experiments [17] are applicable. 

In this study, we aimed to evaluate (i) the in vitro safety of ozone-degrading DON by using HepG2 cells; (ii) the effect of ozone-degrading DON on plant germination; and (iii) grain quality if ozone treatments were conducted on DON-contaminated wheat. In brief, toxicity was assessed in vitro by measuring the influence of ozone-degrading DON on the viability and reactive oxygen index of HepG2. Moreover, quantification of plant seed germination revealed toxicity changes if DON was degraded by ozone. Collectively, the above two methods can objectively evaluate the safety of DON after in vitro degradation, and provide a theoretical basis for safety guidelines. 

## 2. Results 

### 2.1. Effects of DON Degradation on HepG2 Activity 

**Figure 1:**
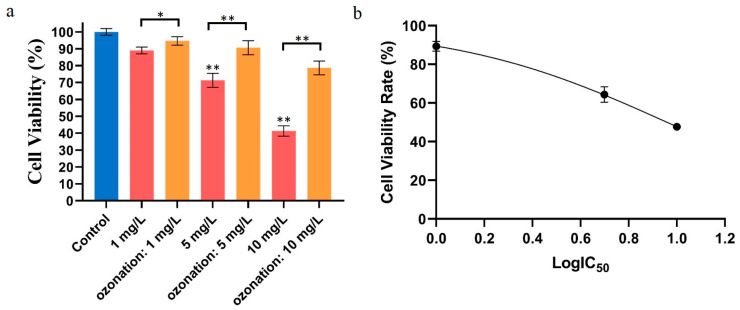
Effects of DON and ozone-treated DON on cell activity. (**a**) Effects of DON and ozone-treated DON on HepG2 cell activity. HepG2 cell viability (%) with exposure to ozonation DON in comparison with that of control cells (blank reagent), as evaluated by the MTT assay. Blue: control group, red: groups directly treated with different concentrations of DON, orange: groups prepared by exposure to ozone-treated DON. (**b**) Modelling curve (nonlinear regression) used to calculate the IC_50_. For each panel, a one-way ANOVA test followed by Tukey’s multiple comparison test was used for statistical analysis of difference between any two groups. All data were acquired from at least three replicates and expressed as mean ± standard deviation. *: *p* < 0.05, **: *p* < 0.01.

In this experiment, HepG2 cells were exposed to different concentrations of DON or ozone-treated DON. As shown in Figure 1a, with increasing concentrations of mycotoxin, the cellular viability rate was more inhibited. When the concentration of DON was 1 mg/L, the HepG2 cell viability rate was about 87%. The cell viability rate decreased to 41.3 % when the exposure concentration was 10 mg/L. As a result, an IC_50_ of 9.1 mg/L was calculated (Figure 1b) using nonlinear regression (Curve fit) methods and GraphPad Prism. However, the viability rate of cells was 94.7% and 90.6% at 1 and 5 mg/L of ozone-treated DON. Due to incomplete degradation of DON toxins by ozone (quantified in our previous study [11]), the cell viability rate was 78.6% at 10 mg/L compared to the control. Additionally, the experimental results showed that DON toxin had a significant inhibitory effect on HepG2, while ozone-degrading DON had no significant effect.

### 2.2. Effects of DON Ozonation on Reactive Oxygen Species in HepG2 

**Figure 2:**
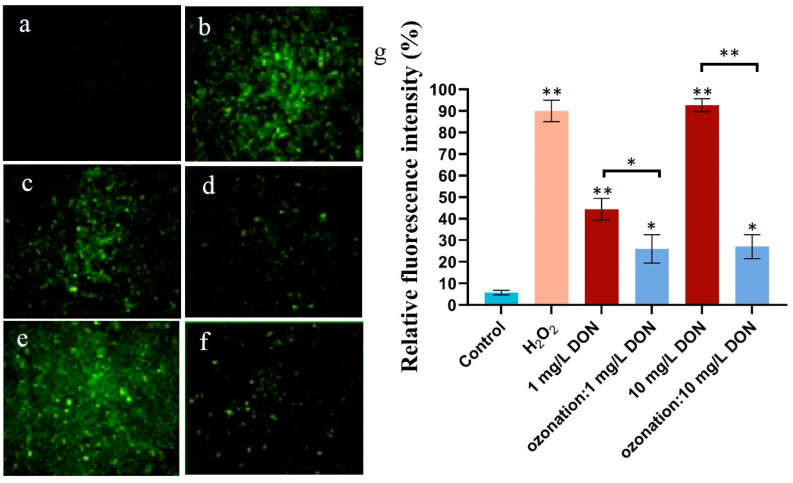
The effect of DON degradation on reactive oxygen species in HepG2 cells. (**a**) Control group; (**b**) H_2_O_2_ treatment group; (**c**,**d**) groups treated with 1 mg/L parental DON or ozone-treated DON; (**e**,**f**) groups with 10 mg/L parental DON or ozone-treated DON; (**g**) relative fluorescence intensity of cells. Light blue: control group, pink: group treated with H_2_O_2_, red: groups treated with different concentrations of DON, blue: groups prepared by exposure to ozone-treated DON. For each panel, the one-way ANOVA test followed by Tukey’s multiple comparison test was used for statistical analysis of difference between any two groups. All values are expressed as mean ± standard deviation of three replicates. *: *p* < 0.05, **: *p* < 0.01.

Cell metabolism produces a variety of oxidative free radicals (ROS, reactive oxygen species) that are continuously cleared in a dynamic balance. When cells are invaded or stimulated, this balance of the antioxidant system is disrupted [18]. The excessive production of ROS in cells will lead to lipid peroxidation, changes in the cell barrier, and abnormal permeability of the mitochondrial membrane and cell membrane, resulting in protein denaturation and DNA breakage [4]. ROS can be measured using DCFH by detecting the fluorescence intensity (Figure 2a,b). As shown in Figure 2, with the increasing concentration of 1–10 mg/L of DON on HepG2, the intracellular ROS increased. Due to incomplete degradation of DON by ozone, the degradation rate reached 83%, while the cell viability rate was 78.6% at 10 mg/L. The fluorescence intensity resulting from 10 mg/L DON was higher than that from 1 mg/L DON (Figure 2c,e). The fluorescence intensity of cells of ozone-treated DON samples was significantly lower than that of parental DON (Figure 2c–f), indicating that the toxicity of DON after degradation was significantly reduced.

### 2.3. Effect of Ozone Degradation of DON on Mung Bean Germination 

In this experiment, DON was extracted and purified from contaminated wheat. The purified DON (2 mg/L) was then treated with ozone at a concentration of 80 mg/L for 10 min. A comparative experiment using DON and degrading DON was thereby conducted upon mung bean germination.

**Figure 3:**
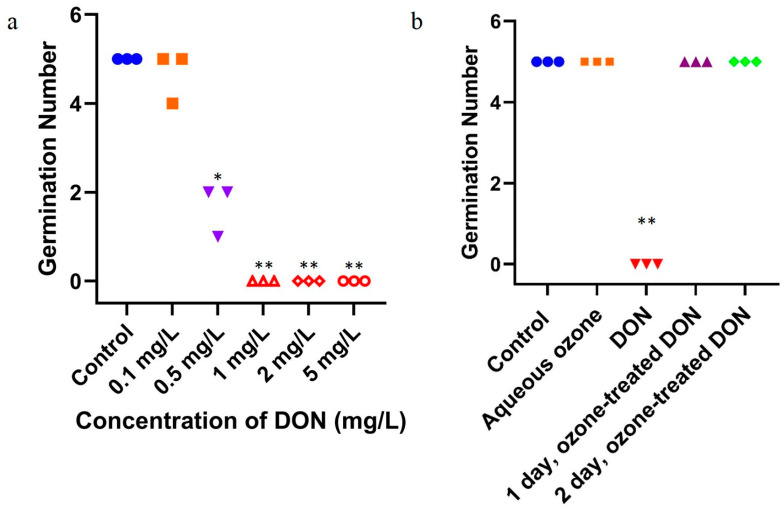
Effects on germination of mung bean after exposure to DON and ozone-treated DON. (**a**) DON exposure; blue circles: control group, orange squares: 0.1 mg/L DON group, purple inverted triangles: 0.5 mg/L DON group; red hollow triangles, diamonds and circles: 1, 2, 5 mg/L DON, respectively. (**b**) Ozone-treated DON exposure; blue circles: control group, orange squares: aqueous ozone group, red inverted triangles: 2 mg/L DON group, purple triangles: the 1-day group of ozone-treated 2 mg/L DON, green diamonds: the 2-day group of ozone-treated 2 mg/L DON. All values are based on three replicates. For each panel, the one-way ANOVA test was used for statistical analysis of difference between any two groups. *: *p* < 0.05, **: *p* < 0.01.

As shown in Figure 3a, compared with the control group without DON, the germination and growth of mung bean were not significantly inhibited when the concentration of DON increased from 0 to 0.5 mg/L, but were significantly inhibited when the concentration of DON increased to ≥1 mg/L. The results showed that high concentrations of DON obviously inhibited the germination of mung bean seeds. Low concentrations of DON (0.1 mg/L) do not inhibit mung bean germination, but high concentrations of DON (≥0.5 mg/L) could inhibit mung bean germination. 

As shown in Figure 3b, the germination and growth of mung bean were significantly inhibited by ≥1 mg/L DON. Compared with the control group, the germination and growth of mung bean were not significantly inhibited if DON was degraded by ozone. 

DON extracted from wheat had a significant inhibitory effect on the germination of mung bean seeds, while DON treated by ozone had no significant inhibitory effect, indicating reduced toxicity. 

Ozone-treated DON in mung bean seeds did not inhibit their germination, as observed on both the first and the second day (Figure 3b). This could imply that ozone degradation can result in attenuated toxicity of DON. Meanwhile, ozone itself did not inhibit seed germination. 

### 2.4. Effect of Ozone Degradation of DON on Wheat Quality

The effects of ozone treatment were investigated for wheat nutrients. As shown in Table 1, compared with the untreated group, the protein of the wheat was not significantly affected by the treatment of 80 mg/L ozone for 1–10 min. There was no significant difference in starch content between the untreated group and the treated group (ozone exposure time: 1 min). The starch content decreased slightly after 10 min exposure, although this change was not significant. Similarly, no significant effect of ozone treatment was observed on the fatty acid in wheat. Nevertheless, no significant differences were found in the protein contents, fatty acid values, and starch contents of ozone-treated wheat samples. 

**Figure 4:**
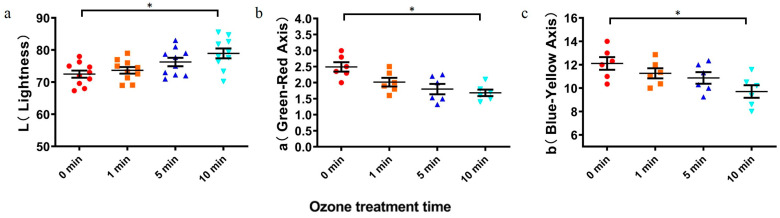
Color changes of ozone-treated wheat flour. (**a**): L, lightness; (**b**): a, green–red; and (**c**): b, blue–yellow. Red circles: 0 min group, orange squares: 1 min group, navy triangles: 5 min group, blue inverted triangles: 10 min group. All values are expressed as mean ± standard deviation of three replicates. For each panel, the one-way ANOVA test followed by Tukey’s multiple comparison test was used for statistical analysis of difference between any two groups. *: *p* < 0.05.

Color is an important indicator of wheat quality. Spectrophotometry was used to assess the color of wheat with ozone treatment. Figure 4 shows the effect of different ozone treatment times on wheat color. During the treatment time, the brightness value of ΔL increased gradually, indicating that the color of wheat flour after treatment was lighter than that of the standard sample. The main color of wheat is due to carotenoids, with orange carotene and yellow lutein. After treatment, the values of a and b were lower than those of the control group, indicating that the yellowness value of wheat decreased slightly after treatment. Similar to the results of Li et al., our observation showed increased brightness and whiteness values of grains if wheat was treated by ozone [15]. Moreover, the ozone-treated samples exhibited higher tenacity and whiteness, as well as lower extensibility and yellowness (Figure 4). Thus, ozone degrades the toxin in wheat, with little effect on quality.

## 3. Discussion

Previously, ozone has been applied to the degradation of DON in wheat. However, the contaminated wheat’s safety and quality were often ignored after ozone degradation. DON may accumulate in the food chain, increasing the exposure risk of toxic DON [3]. Therefore, implementation approaches for assessing the safety of DON-contaminated or ozone-treated DON-containing grain are of great significance for food safety.

In this paper, we performed an evaluation by using in vitro cell experiment and by measuring wheat quality, revealing the potential safety of ozone-treated DON. In this study, we used HepG2 cells as an in vitro model to investigate the potential damage from DON-induced cytotoxicity. The HepG2 cell and mung bean seed germination experiments were applicable to in vitro safety evaluation; we did so by comparing the groups treated with parental/extracted DON and ozone-degrading DON. We determined that the IC_50_ was 9.1 mg/L, being associated with the cytotoxicity of DON in HepG2 cells. In addition, DON-treated groups showed a dose- and time-dependent decrease in cell viability. HT-29 cells became sensitive to DON at 48 h, with an IC_50_ of 4.72 mg/L [19]. Further, the MTT assay found that HepG2 cells exposed to DON for 48 h had an IC_50_ value of 12.3 mg/L [20]. These serial differences related to the IC_50_ concentration of DON might result from the susceptibility of the cell lines. Remarkably, DON resulted in increased production of intracellular ROS, while ROS decreased significantly if ozone pre-treatment was conducted on DON [21,22]. However, the inhibition rate of HepG2 cells was significantly lower if DON was degraded by ozone. With the increase in DON concentration, the damage to cells was more significant [23].

The above results could suggest that the toxicity of DON-contaminated wheat was significantly reduced after ozone degradation. They imply that the transformation of DON toxin by ozone water might be irreversible. In future, we will quantify the sustainability and effectiveness of DON degradation by ozone water [24].

Wheat is commonly highly contaminated by DON. We extracted and purified DON in wheat, to examine the effects of DON on the germination of mung bean seeds. Interestingly, there was no inhibition if ozone-degrading DON was added to mung bean, com-pared to the inhibitory effects driven by prepared DON. We assumed that ozone could effectively degrade DON toxins in contaminated grains, resulting in significantly reduced toxicity to plant seeds [25]. Then, we determined the impact of DON degradation by ozone on wheat quality. No changes in wheat quality related to total protein, fat, and starch contents were observed. The color of wheat was slightly affected by ozone treatment. In the context of DON degradation, ozone treatment is feasible for food safety and storage. Our study indicated that the toxicity of wheat contaminated with DON decreased significantly after ozone degradation. Taken together, this work provided new insights into the safety and quality assessment of the DON-contaminated grains. 

## 4. Conclusions

In summary, our finding indicated that DON treated by ozone on wheat could reduce its toxicity. With a short-time ozone treatment, there was little effect on the quality of wheat. Thus, the ozone treatment efficiently degrades this toxin in wheat, with little negative effect on grain quality. Prospectively, this ozone degradation technology has potential for DON control. 

## 5. Materials and Methods

### 5.1. Chemicals and Materials

Standard DON, acetonitrile, and methanol were purchased from Sigma-Aldrich Corp. (St. Louis, MO, USA). The standard DON was dissolved in methanol at concentrations ranging from 1 to 20 mg/L and stored at 4–6 °C. For the HPLC detection of the samples, reagents of chromatographic purity were used to dissolve samples and through a 0.22 μm filter. The others were of analytical grade. 

### 5.2. Extraction of DON from Wheat

In this study, we extracted DON from wheat for mung bean germination experiments, and we used DON standard chemicals for experiments of HepG2 Cells.

As a result, DON was extracted from DON-contaminated wheat for our experiment on the Inhibition of DON in Mung Bean Germination. This test could provide a supportive basis for using ozone degradation technology to control DON in contaminated wheat. We extracted DON from DON-contaminated wheat according to the China National Standard (GB/T 23503-2009) [26]. 

### 5.3. Culture of HepG2 

HepG2 cells were obtained from the Cell Bank of Chinese Academy of Sciences (Shanghai, China). HepG2 cells were archived in a liquid nitrogen tank. We then quickly thawed and incubated them at 37 °C [4]. These cells were centrifuged at 1000 r/min for 5 min and the supernatant was discarded. Next, we added an appropriate amount of culture medium to resuspend the cells and centrifuged again to remove the supernatant. Then, we added 5 mL of bovine serum and 45 mL of complete culture medium made from DMEM, and performed sufficient resuspension to prepare a mixture. Then, we transferred the cells to a culture bottle and placed the bottle in the incubator for cultivation. Of note, the culture medium was renewed every 3 days. When the cell coverage area at the bottom of the bottle reaches 90%, subsequent culture can be carried out. First, we washed the cell bottle twice with PBS (phosphate-buffered saline), and then added trypsin for digestion for 2 min. Next, 2 mL of protease K was added to terminate the digestion reaction. We centrifuged it again at 1000 r/min for 3 min, and discarded the supernatant. Finally, the cells were resuspended using a complete culture medium and divided into bottles at a ratio of 1:2. The HepG2 cells were usually subcultured every 3–4 days. To maintain individual homogeneity, the number of passages should be <20.

### 5.4. MTT Testing of HepG2 Activity

The MTT method is a common test used to determine the proliferation activity of cells. Dehydrogenase in the mitochondria of living cells can reduce yellow tetrazolium salt into a blue–purple crystalline compound. This compound is deposited in cells, while dead cells do not have this ability. Formazan can be dissolved in dimethyl sulfoxide (DMSO) and then the optical density (OD, absorbance value) can be determined using a microplate reader. The OD value is directly proportional to the number of living cells, which can indirectly reflect the change of living cells, allowing calculation of the inhibition rate of toxin on cell viability during cell culture.

Through an MTT assay, the growth inhibition effect of different concentrations of DON on HepG2 cells can be evaluated. By measuring the absorbance value (460 nm), the inhibition rate can be calculated to evaluate the toxicity [19]. The following are the steps for MTT testing [20]: HepG2 human liver cancer cells were obtained in the logarithmic growth phase and prepared into a suspension through digestion and centrifugation. We adjusted the cell concentration and inoculated 5 × 10 ^3^ cells/well into a 96-well plate. We cultivated cells for 24 h, until the cells adhered to the wall. Then, we carefully removed the culture medium and added raw culture medium containing different concentrations of DON (toxic substances). Different concentrations of DON were prepared, such as 1, 5, and 10 mg/L. Meanwhile, normally cultured HepG2 cells were used as the control group. For the treatment group, culture media prepared with parental DON or ozone-degrading DON were added separately. DON toxin was pre-dried with nitrogen gas and added to the culture medium for re-dissolution. Six parallel dose groups were customized, with 200 μL DON or its derivative added to each sample before incubation in a CO_2_ incubator for 24 h. After discarding the supernatant, we added 100 μL MTT solution to each well with a concentration of 1 mg/mL, followed by 4 h cultivation. The supernatant was carefully removed and we added 150 μL DMSO (solvent) to each well, shaking for 10 min. The absorbance (OD) value was measured at 460 nm. 

### 5.5. Determination of Intracellular Reactive Oxygen Species Levels in HepG2

The measurement of intracellular reactive oxygen species levels is carried out using DCFH-DA (2′,7′-dichlorodihydrofluorescein diacetate) as a reactive oxygen species detection agent. Fluorescent dye-based intracellular ROS detection was performed using 2′,7′-dichlorodihydrofluorescein diacetate (DCFH-DA; Invitrogen Corporation, Carlsbad, CA, USA) as previously reported. Relative fluorescence intensity was measured using a SpectraMax M2 microplate reader (Molecular Devices, Sunnyvale, CA, USA) with predefined excitation/emission wavelengths of 488/525 nm [27]. 

### 5.6. Inhibition Experiment of Ozone-Degrading DON on Mung Bean Germination 

The experimental groups were as follows: (1) the blank group, treated with distilled water; (2) the control group, parental DON groups with serial concentrations of 0.1, 0.5, and 1 mg/L of DON; and (3) the dosage group, ozone-degrading DON groups with concentrations of 1, 2, and 5 mg/L of DON derivative. The germination conditions for mung beans were as follows. Beans were purchased from the supermarket, and then were divided into different groups for soaking 12 h, and beans were filtered and wrapped in gauze. We incubated the beans at a constant temperature of 37 °C for 24 h to observe the germination of mung beans [14,15]. 

### 5.7. Determination of Ozone Degradation of DON and Its Effect on Wheat Quality

WSC-S-based colorimetry was used to measure the color of wheat flour [28]. The CIE-Lab* color space was used to represent the chromaticity of wheat flour, where ΔL* represents the brightness, a* represents the red–green value, and b* represents the yellow–blue value. The determination of protein content in wheat was achieved as follows. The Kjeldahl method was used to determine the protein content. The starch content in wheat was determined using a starch content test kit (the anthrone colorimetric method). Fatty acid values in wheat flour were determined using an acidity meter. 

### 5.8. Statistical Analysis

GraphPad Prism 8.0 (GraphPad Software, San Diego, CA, USA) was used to perform the statistical analysis of the data. The data are expressed as mean ± standard deviation of different independent experiments. Differences among groups were analyzed using a one-way analysis of variance (ANOVA) unless specifically stated. 

## Figures and Tables

**Table 1:** Changes in nutrient properties of ozone-treated wheat flour.

Ozone Treatment Time (min)	Protein Content (g/100 g·Wheat)	Starch Content (g/100 g·Wheat)	Fatty Acid Content (mg/100 g·Wheat)
0	11.6 ± 0.1	62.5 ± 0.6	52.7 ± 0.4
1	12.1 ± 0.3	62.2 ± 0.3	53.2 ± 0.2
5	12.3 ± 0.5	61.5 ± 0.5	53.5 ± 0.7
10	12.6 ± 0.2	61.1 ± 0.3	53.8 ± 0.3

All values are expressed as mean ± standard deviation of three replicates.

## Data Availability

Data are contained within the article.
